# Variation in bacterial pathotype is consistent with the sit-and-wait hypothesis

**DOI:** 10.1099/mic.0.001500

**Published:** 2024-09-17

**Authors:** Eliza Rayner, Amelie Lavenir, Gemma G. R. Murray, Marta Matusewska, Alexander W. Tucker, John J. Welch, Lucy A. Weinert

**Affiliations:** 1 Dept. Veterinary Medicine, Madingley Road, University of Cambridge, Cambridge, CB3 0ES, UK; 2 Dept. Genetics, Evolution and Environment, University College London, Darwin Building, Gower Street, London, WC1E 6BT, UK; 3 Department of Medicine, University of Cambridge, Box 157 Addenbrooke’s Hospital, Hills Road, Cambridge, CB2 2QQ, UK; 4 Wellcome Sanger Institute, Hinxton, Saffron Walden, CB10 1RQ, UK; 5 Dept. Genetics, Downing Street, University of Cambridge, Cambridge, CB2 3EH, UK

**Keywords:** environmental survival, evolution of virulence, indirect transmission, Pharaoh’s curse, resource allocation hypothesis, *Streptococcus suis*, zoonosis

## Abstract

The sit-and-wait hypothesis predicts that bacteria can become more virulent when they survive and transmit outside of their hosts due to circumventing the costs of host mortality. While this hypothesis is largely supported theoretically and through comparative analysis, experimental validation is limited. Here we test this hypothesis in *Streptococcus suis*, an opportunistic zoonotic pig pathogen, where a pathogenic ecotype proliferated during the change to intensive pig farming that amplifies opportunities for fomite transmission. We show in an *in vitro* environmental survival experiment that pathogenic ecotypes survive for longer than commensal ecotypes, despite similar rates of decline. The presence of a polysaccharide capsule has no consistent effect on survival. Our findings suggest that extended survival in the food chain may augment the zoonotic capability of *S. suis*. Moreover, eliminating the long-term environmental survival of bacteria could be a strategy that will both enhance infection control and curtail the evolution of virulence.

## Introduction

**Fig. 1.**
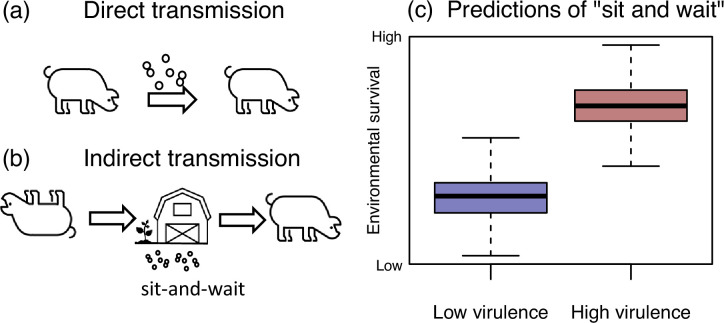
Prediction of the sit-and-wait hypothesis. (a) Direct transmission of a host-associated microorganism relies on a relatively healthy host to transmit, selecting against very high virulence. (b) Indirect transmission could allow for higher virulence, because the fitness costs associated with host ill-health and immobility are relaxed. (c) This leads to a predicted positive correlation between virulence and environmental survival.

Evolutionary theory posits that the virulence of a directly-transmitted microorganism, defined as its effect on host mortality, is subject to a trade-off between increasing within-host replication by exploiting the host, and keeping the host healthy, since both can aid transmission [1,3]. This trade-off is altered by the possibility of indirect transmission via the environment, because environmental survival outside of the host reduces the microorganism’s dependence on healthy hosts for transmission [4,6] (Fig. 1a, b). Together, this leads to the ‘sit-and-wait hypothesis’ (also known as Pharaoh’s curse), which predicts a positive correlation between the virulence of pathogens and their ability to survive outside of their host (Fig. 1c). This implies, for example, that a shift from commensality to pathogenicity might be accompanied by an increase in durability in the environment. The sit-and-wait hypothesis is controversial because the modelling relies on various assumptions [7,10], which may not hold in nature. For example, there may be an energetic trade-off between traits that involve virulence and environmental survival; the ‘resource allocation hypothesis’ makes the opposite prediction between virulence and durability to that shown in Fig. 1c [11]. There is empirical support for the sit-and-wait hypothesis but these are largely limited to comparative meta-analyses [6,12, 13] with few experimental tests. An exception is an empirical study that showed induced starvation resulted in both increased and decreased virulence in the bacterium, *Flavobacterium columnare* [14].

The porcine bacterium*, Streptococcus suis* makes a good experimental model to test the sit-and-wait hypothesis due to its host-to-host transmission [15,16], known variation in virulence [17,18] and ability to survive in the environment [19,21]. For example, *S. suis* can survive on farm surfaces, such as dust and faeces for up to 25 days, depending on temperature [21]. *S. suis* is an opportunistic pathogen, carried asymptomatically in the upper respiratory tract of healthy pigs [22,23], but it can also cause severe respiratory and systemic infections and zoonotic infections in humans [24,25]. While *S. suis* cannot transmit from disease sites, virulence factors are thought to aid transmission from carriage sites [26]. For example, the presence of an outer polysaccharide capsule helps the bacterium evade phagocytosis [27] but may also aid shedding and transmission [28]. Moreover, the clustering and expansion of disease isolates in the *S. suis* phylogeny [17,18], implies that pathogenicity is not maladaptive or an evolutionary dead end. There is also suggestive evidence that pathogenicity in *S. suis* co-evolved with changes in opportunities for transmission. Previous work has shown several pathogenic lineages of *S. suis*, one of which is responsible for most zoonotic infections, expanded during the growth of the global pig population and agricultural intensification [17]. These lineages are distinguished by acquisition of three genomic islands that suggest an ecological change in host-pathogen interactions [17] and thus define a pathogenic ecotype. Intensification increases opportunities for fomite (contaminated surface) transmission due to indoor rearing, while higher pig densities could reduce dependency on healthy pigs for transmission [29,31].

Here we test the hypothesis that the pathogenic ecotype of *S. suis* exhibits longer environmental survival than the commensal ecotype. We use a comparative *in vitro* survival assay with a paired experimental design to compare the survival of desiccated cells from pathogenic/commensal isolates on a surface outside of the host. While we observed no consistent difference in rates of population decline between pathogens and commensals, pathogen populations survived for significantly longer than commensals. Our results support the sit-and-wait hypothesis as a plausible explanation for variation in virulence and highlight the potential importance of environmental durability for infections, including zoonoses.

## Methods

### Isolate selection

Our chosen panel of 68 isolates were selected to be representative of pathogenic (*n*=39) and commensal *S. suis* ecotypes (*n*=29) following results from Murray *et al.* [17], which also describes their collection from pigs, and are given with their metadata in Table S1. Pathogenic ecotypes were chosen from genetic clusters with the highest proportion of isolates from disease sites (e.g. blood, brain) relative to carriage sites (e.g. nose, tonsil). Commensal isolates were chosen from genetic clusters with the highest proportion of isolates from carriage sites versus disease sites. In addition, all pathogenic isolates were from disease sites and commensal isolates from carriage sites. Isolates were otherwise chosen to represent the geographic and genetic diversity of *S. suis* (Fig. S1, available in the online version of this article; [18]). A phylogenetic tree was constructed from the core genome using Panaroo v1.2.2 [32] and a distance matrix and neighbour-joining tree was created using the ape v. 5.7-1 package in R [33] (Fig. S1). Polysaccharide capsule serotypes were determined *in silico* using the Athey *et al.* [34] pipeline.

### Environmental survival assay

Frozen glycerol stocks were streaked on Columbia sheep blood agar plates (Fisher Scientific, UK) and incubated overnight at 37 °C in a static incubator. Single colonies were used to inoculate 10 ml Todd Hewitt Broth with 2% yeast extract (THB+Y) and incubated overnight at 37 °C in a static incubator. For the environmental survival assay, the overnight culture was adjusted to concentrate 10^9^ colony forming units (c.f.u.) in 10 µl using optical density measurements at 600 nm wavelength (accounting for clade-specific c.f.u. ml^-1^ to OD600nm correlations). This 10 µl suspension was spotted into 12 wells of a 96-well plate and left to air dry for 150 min. Six isolates were tested per plate, alternating rows between pathogens and commensals to eliminate any effect of position in the 96-well plate on survival and ensure biological consistency between the two clinical categories in the experiment. Then 10 µl sterile THB+Y was pipetted into all wells of the top row to act as a negative control, while *S. suis* P1/7, a laboratory strain that was first isolated in 1976, was used as a positive control in the bottom row of each plate (an example plate is shown in Fig. S2A). Immediately after the 96-well plates had dried, 100 µl THB+Y was pipetted into all eight wells of the first column of the 96-well plate. A pipette was used to mix the contents of the wells thoroughly for 30 s. The 100 µl broth was transferred into 900 µl PBS and enumerated through seven serial dilutions (from 10^−1^ to 10^−8^). This measurement is marked at time 0 in Table S2. The 96-well plate was covered with AlumaSeal foil film (Merck, Germany) and was incubated at room temperature for the duration of the experiment. Because measurement of a well destroyed the colonies in that well, we could not track individual wells at multiple different timepoints. For this reason, our design was to spot multiple wells with the same isolate, and then measure each well at a single, predetermined timepoint (Fig. S2A, B). There was variation in sampling days between plates (see Table S2), but each pathogen/commensal comparison was measured over the same timescale. The thirteen plates were grouped in to five batches where the experiments were conducted over the same month (Fig. S3). Full details of assignment of isolates to plates, and of plates to batches are given in Tables S1 and S2.

Measurements of the remaining viable cell count from subsequent wells (in the same manner as above) were taken over time until no growth was visible (Fig. S2). For these measurements, colonies were counted after 72 h incubation to allow for the delayed growth of colonies. When the number of colonies observed at the 10^−1^ dilution decreased below ten, 10 µl of the 100 µl broth pipetted into the well was spotted directly onto a THB+Y agar plate to enable the detection of lower cell counts. Raw data is given in Table S2.

### Statistical analysis

For each isolate, we obtained estimates of total c.f.u. at a range of different timepoints, which formed a time series (albeit obtained from independent replicates, rather than the tracking of a single population over time). Environmental survival for each isolate was estimated in two ways, as illustrated in Fig. S2B. First, we defined ‘survival time’ as the last day that viable cells could be detected, following 3 days of incubation at 37 °C. This statistic was therefore bounded above at the last day that was measured for a given batch of plates. Therefore, some isolates survived until the end of the experiment (Fig. S2B). Second, we defined the ‘rate of population decline’ as the best-fit regression slope of c.f.u. onto time (removing initial starting c.f.u.), using Poisson regression and a log link function. The use of the log scale implies a measure of proportional population decline. The Poisson regression failed to converge for two strains, which therefore lacked estimates of this second statistic. We note that our two measures are expected to be highly correlated in theory, because populations that decline more slowly are expected to survive longer. However, biologically, dynamics may depart from exponential decline at longer times, while statistically, estimates of the decline rate will also be dominated by the initial generations. As such, the two statistics may capture different aspects of environmental survival. All statistical tests were carried out in R v.4.2.2.

## Results

### Pathogens have a longer survival time than commensals

**Fig. 2.**
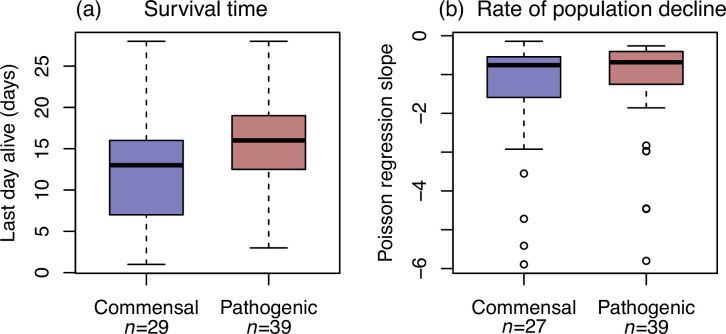
Pathogens have longer survival times than commensals but no consistent difference in rates of population decline. Box plots showing (a) survival time (last day alive) and (b) rate of population decline for a panel of commensal and pathogenic ecotypes of *S. suis*. Boxes and whiskers show first and third quartile and points show outliers. Rate of decline was estimated by Poisson regression, which did not converge for two commensal isolates (see Table S2).

Fig. 2a shows the survival times (last day alive) of the two groups of isolates. There is a clear difference between the commensal and pathogenic *S. suis*, which is consistent with the predictions of the sit-and-wait hypothesis (Fig. 1c). However, the results plotted are likely to be confounded by differences in experimental conditions between batches of plates that were analysed over the same period, or between plates within a batch. Indeed, Fig. S3 shows that there was biological variation for survival time, both within and between batches, in our positive control strain, that was included on all plates. However, variation for all isolates was greater between than within batches (e.g*.* survival time between: SD=5.86; within: SD=3.42), consistent with environmental parameters (e.g. temperature) explaining some of this variation.

As such, we considered a paired design, comparing the mean survival times for commensals and pathogens within batch or plate. As shown in Fig. S4A, results were nonetheless consistent with sit-and-wait. In all 12 plates containing both commensals and pathogens, the pathogens tend to have longer survival times (Fig. S4A; median extra survival days=4.95; exact binomial two-tailed, *P*<0.001). The same was true of all five batches of plates (Fig. S4).

### No consistent difference in rate of population declines between pathogens and commensals

In contrast, when modelling daily decline in colony forming units (c.f.u.; see Methods), there was no consistent difference in the rate of population decline between pathogens and commensals. This is true of the raw data (Fig. 2b), but also of the paired test within batches or plates (Fig. S4B; exact binomial two-tailed; *n*=12, *P*=0.227). Our results imply that while the rate of decline is not consistently different between pathogens and commensals, pathogens tend to persist over time.

### Results are not due to differences in initial population size

Another possible source of confounding is differences in starting c.f.u. between pathogens and commensals (since larger initial populations are likely to survive longer). While we aimed to aliquot 10^9^ c.f.u. into each well on each plate, our actual c.f.u. differ between by an order of magnitude in either direction (Fig. S3C). However, this does not affect our results. We find that while the starting c.f.u. is highly correlated to the c.f.u. immediately after desiccation (Fig. S5A), it did not significantly correlate with rate of population decline (Fig. S5B). There was a weak positive correlation between starting c.f.u. and survival time, but this was due to four isolates that had the lowest c.f.u. values (Fig. S5C). Our survival time results are robust to excluding these four isolates (see white circles in Fig. S4A). Moreover, pathogens tended to have a lower starting c.f.u. on 8/12 plates (Fig. S4C), which suggests that starting c.f.u. cannot explain the observed difference in survival times.

### No consistent effect of capsulation on survival times

Our pathogenic isolates all have polysaccharide capsules, while there was variation in capsule presence in commensals (Table S1). The desiccation resistance and energy reserves that capsules provide is a plausible mechanism for increasing survival time [35,36]. However, when considering commensal isolates alone, there was no consistent effect of possessing a capsule on overall survival times (Fig. S6). There is insufficient data to test the effect of individual capsule types or serotypes.

## Discussion

Our study suggests that the sit-and-wait hypothesis may explain variation in pathogenicity within *S. suis*. Pathogenic isolates showed longer environmental survival compared to commensal isolates. This is also consistent with meta-analyses showing that pathogens, including opportunistic pathogens like *S. suis*, have longer survival times [6]. Our results also align with predictions by Roche *et al.* [10] where they find two stable virulence strategies driven by environmental factors. However, this study assumed a resource allocation trade-off between virulence and environmental survival, which is counter to our evidence. This may be because traits like the polysaccharide capsule can play a pleiotropic role in survival in both environments [35,37].

Polysaccharide capsules are more abundant in pathogenic ecotypes and have previously been shown to enhance environmental protection in other bacteria [35,36]. Our results do not show support for presence of the capsule enhancing survival time. However, this does not negate a role for a specific capsule type or capsule expression explaining longer survival times of our pathogenic ecotype. It is notable that pathogenic and commensal ecotypes have different capsule types (with serotypes 1, 1/2, 2 and 14 only found in pathogenic ecotypes; Table S1). On the other hand, our observation that rates of decline are similar between ecotypes but that pathogenic ecotypes survive for longer, could show that they have a higher propensity or ability to enter a ‘persister’ cell state. Persister cells are durable subpopulations of bacteria that have undergone a reversible phenotypic switch into a metabolically dormant state that confers antibiotic tolerance [38]. Persisters have previously been identified in *S. suis* [39] and can form in response to stresses experienced during outside-host survival such as nutrient deprivation [40,42]. Whether or not they involve persisters, our results might also be caused by phase variable genes. Such genes have been shown to impact growth in *S. suis* [43], and could be more abundant in pathogenic ecotypes [44].

Our findings may also have translational implications. Disinfection methods targeting extended survival outside the host could not only enhance infection control, but also limit the evolution of more virulent strains. Many pig production systems are all-in-all-out, with disinfectant procedures to limit disease transmission between batches. The time between de- and re-population can vary but 1 week is not unusual [45] and is relevant to the timescale detected in this study. Furthermore, though chosen on the basis of virulence in pigs, most pathogenic strains tested were from a subgroup associated with zoonotic disease [17,46]. This indicates that extended survival in the food chain may increase the risk of zoonotic transmission.

Our study has notable limitations. The pathogenic ecotype emerged from the commensal ecotype and therefore our correlation suffers from phylogenetic non-independence (Fig. S1). Future support testing the sit-and-wait hypothesis in other systems should target systems that show multiple independent virulent emergence events, although these systems will be rare in practice. In addition, our experiment was performed under desiccation in laboratory conditions and conditions on farms and in the food-chain will be far more variable with respect to environmental parameters such as temperature, pH and humidity and the presence of other microorganisms.

## Conclusion

Pathogenic ecotypes of *S. suis* showed greater environmental durability than commensal ecotypes after prolonged periods of survival outside of their hosts. This study represents a novel approach to empirically testing the sit-and-wait hypothesis and is the first to investigate within-species pathotype variation. Our results suggest that eliminating the long-term environmental survival of *S. suis* is important in reducing its agricultural and zoonotic transmission and may help to suppress further evolution of virulence in *S. suis*.

## supplementary material

10.1099/mic.0.001500 Uncited Supplementary Material 1.

10.1099/mic.0.001500 Uncited Supplementary Material 2.

10.1099/mic.0.001500 Uncited Supplementary Material 3.

## Abbreviations

c.f.u.: colony forming units
PBS: phosphate buffered saline
SD: standard deviation
THB+Y: Todd Hewitt Broth with 2% yeast extract
