# Supplementary material for: Variation in bacterial pathotype is consistent with the sit-and-wait hypothesis
Source: Microbiology (Reading). 2024 Sep 17;170(9):001500. doi: 10.1099/mic.0.001500 (PMC11407517; doi:10.1099/mic.0.001500)
Supplement: Uncited Supplementary Material 1. [file mic-170-01500-s001.pdf]

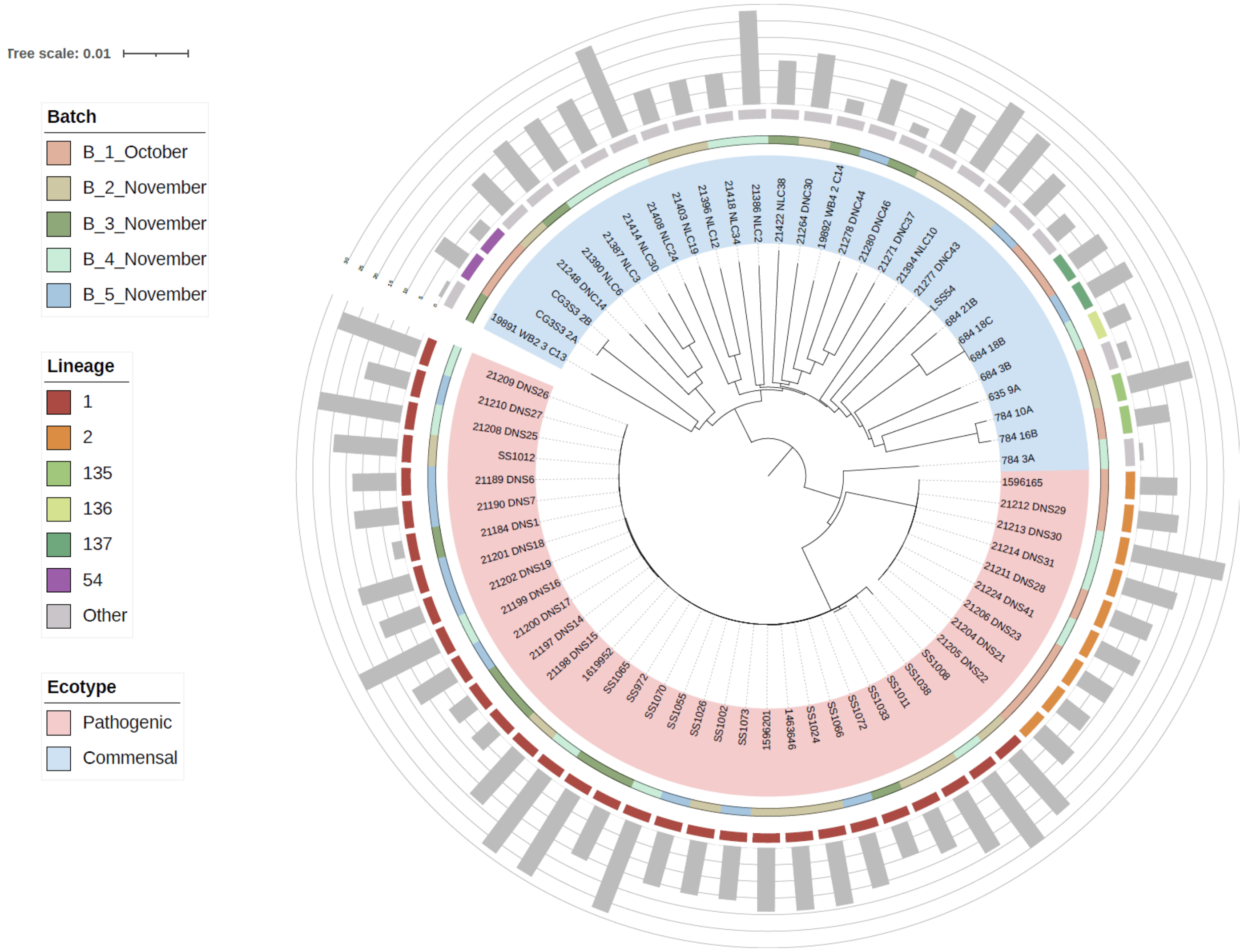

Figure S1. Neighbour joining tree of isolates used in this study. The tree was pruned from a phylogeny of 3,070 isolates taken from Murray *et al.* (20). The tree was constructed from the core genome using *Panaroo* v1.2.2 (45) and a distance matrix and neighbour-joining tree was created using the *ape* v. 5.7-1 package in R (46). Clade colours refer to the two different ecotypes and tip labels to experimental batch and genetic lineage taken from Murray *et al.* (20). The last day alive of each isolate is given as a barplot on the outside of the tree with each line representing five days.

A

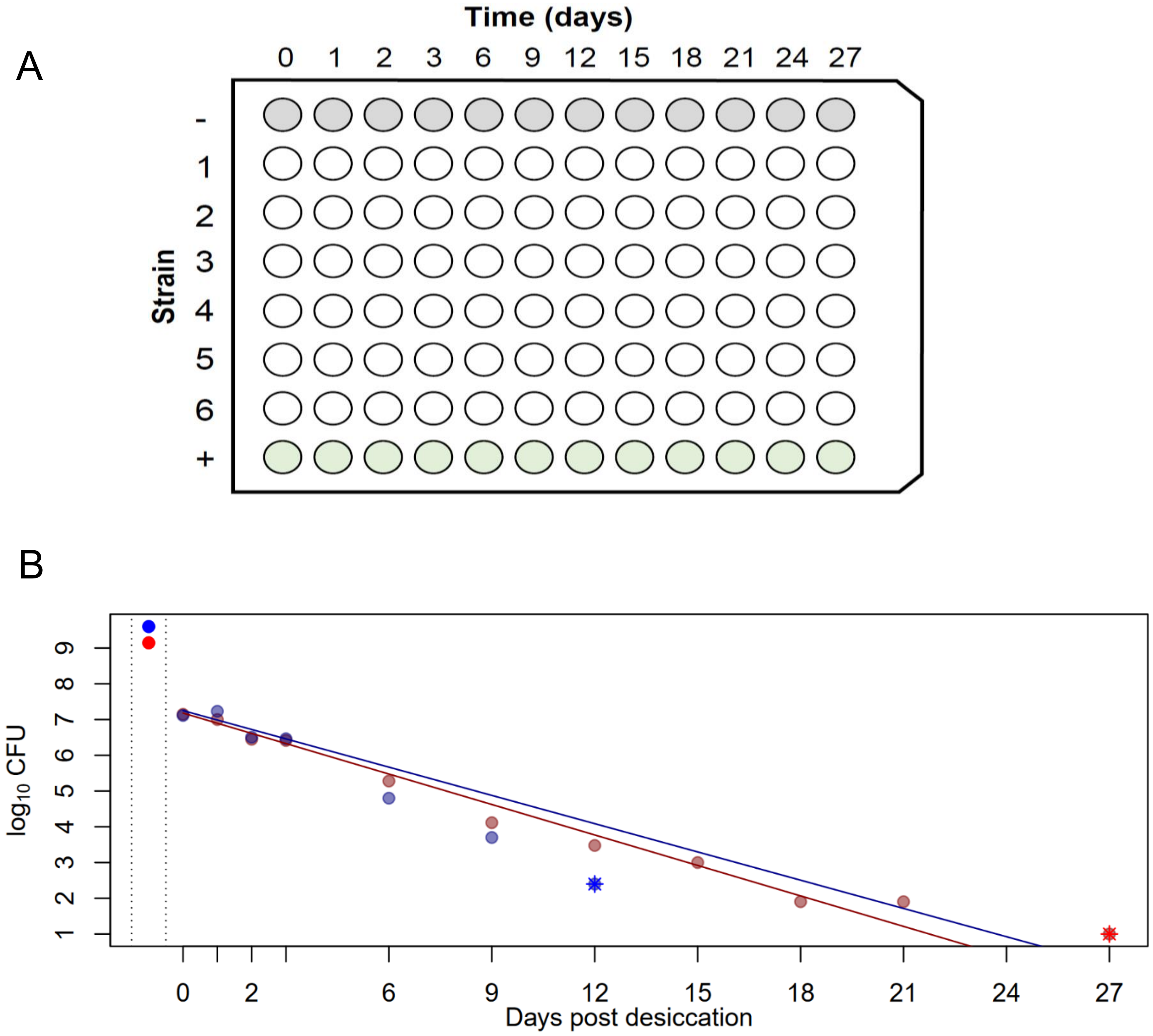

Figure S2. Schematic of study design and data collection. A. Each plate had *Streptococcus suis* P1/7 as a positive control and PBS as a negative control. Pathogenic and commensal ecotypes alternated between rows. Each well was measured at a single pre-determined timepoint as indicated in the column labels. The rates of decline and survival time (i.e. last day alive) for each isolate were summary statistics of measurements of a single row of wells. B. Example results are shown for two isolates from plate 6 in Batch 3. The two isolates are NLC38 (commensal; blue points) and SS972 (pathogenic; red points). CFUs were measured before desiccation, and this initial CFU is shown as the brighter points on the left-hand side of the plots. For this batch, CFUs were measured at 12 different timepoints post-desiccation, corresponding to the 12 wells on each row of the plate (Figure S2A). Our two statistics, used to quantify environmental survival were the survival time (defined as the last day alive, and shown as the asterisks), and by the rate of decline (defined as the best-fit slope of a Poisson regression of CFU on days post desiccation. Results show that the former statistic is bounded above by the last day measured (27 days post desiccation in this case), and that the latter statistic is more strongly influenced by the earlier generations, when population sizes are larger. This means that the two statistics capture partially independent aspects of environmental survival.

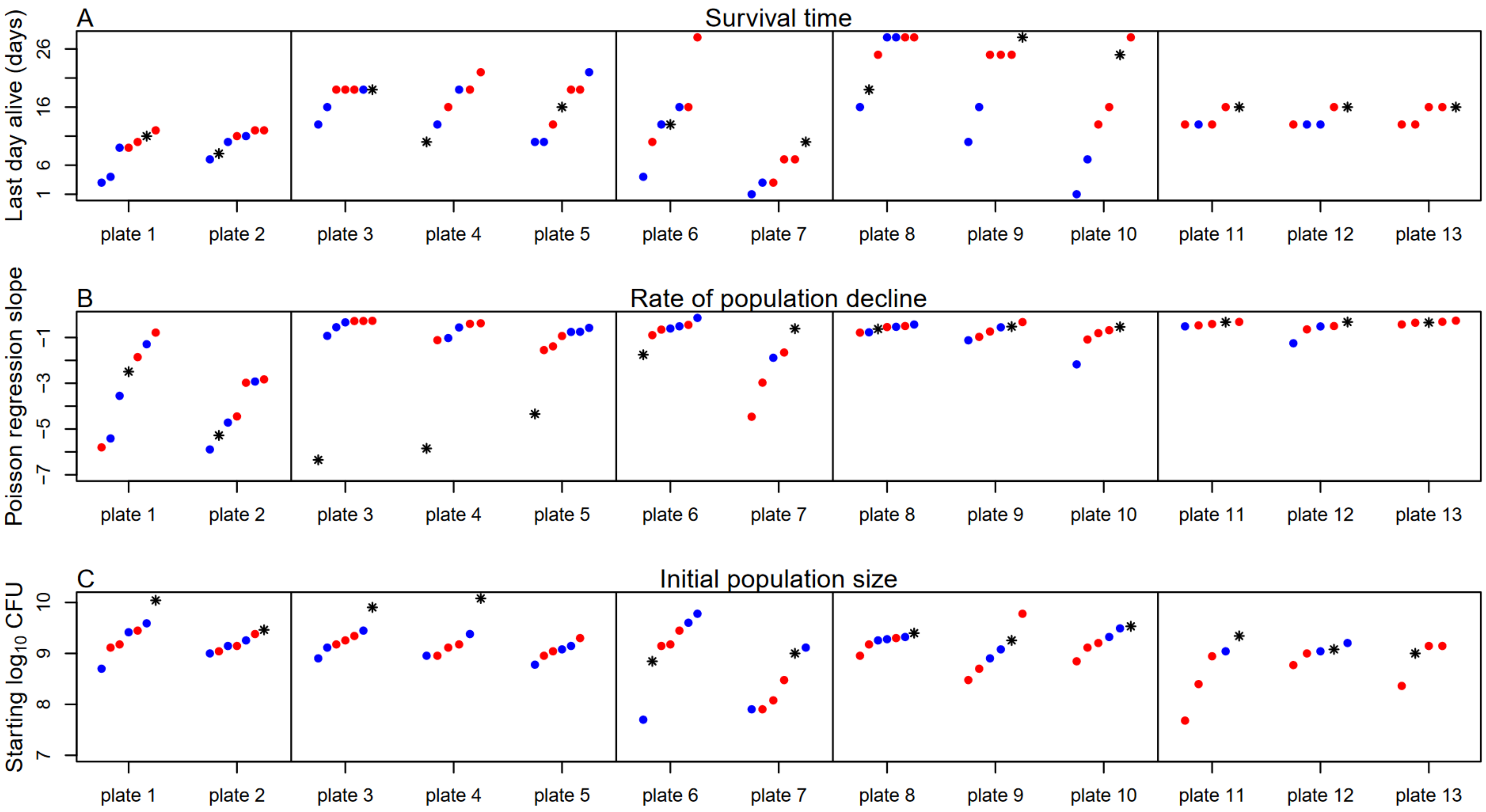

Figure S3. Experimental measurements. A. Survival times (days) for each plate of isolates. B Rates of population decline C. The estimated numbers of colony forming units (CFU) spotted on to the plates. In all cases, points are ordered by measurement low to high, not by their order on the plate. Pathogens are red circles, commensals are blue circles and the positive control strain (P1/7) is indicated by black asterisks. Black vertical lines demarcate each batch of plates that were measured over the same time period (left-to-right batches 1-5).

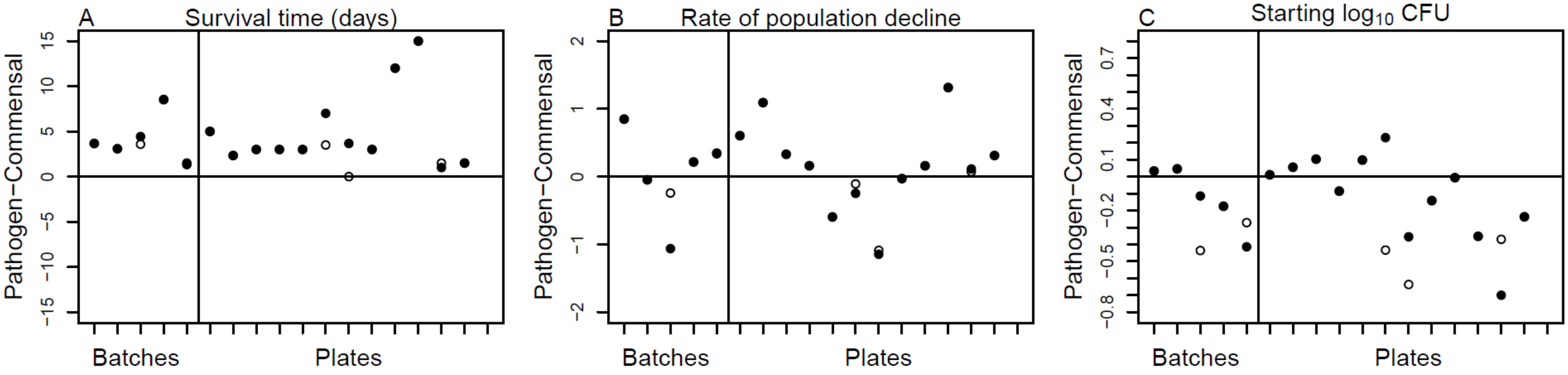

Figure S4. Pathogens have longer survival times than commensals but no consistent difference in rates of population decline or starting CFU. A. Difference in survival time (days) between pathogens and commensals. Above zero: pathogen survival times are longer; below zero: commensal survival times are longer. B. Difference in rates of population decline between pathogens and commensals. C. Difference in starting CFU between pathogens and commensals. Black solid circles show the difference in means for all pathogenic or commensal isolates within a single batch or plate. White open circles show the equivalent statistic excluding isolates with low initial starting CFU (i.e. the isolates shown as starred points in Figure S5C). Plates are grouped into batches that were measured over the same time of year, and follow the ordering used in Figure S3.

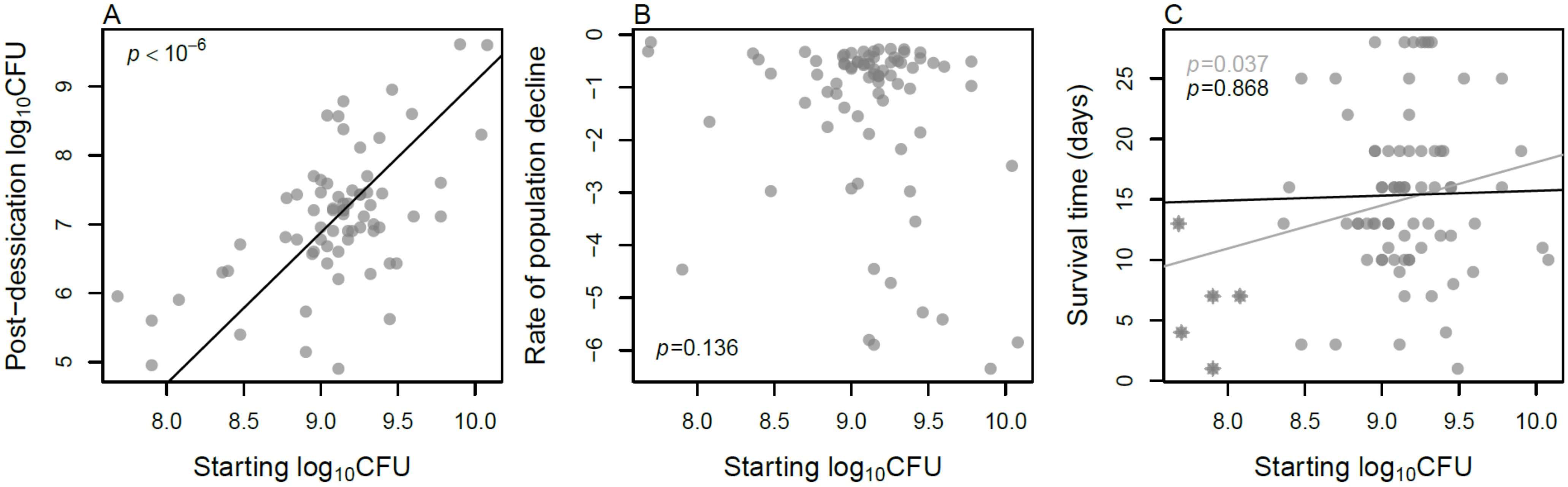

Figure S5. The influence of starting numbers of bacterial cells. Correlations between colony forming units (CFU) and A. Post-desiccation CFU, B. Rates of population decline and C. Survival times (days). The correlation in plot C is not significant (p=0.86) when removing points with low starting CFU (starred points).

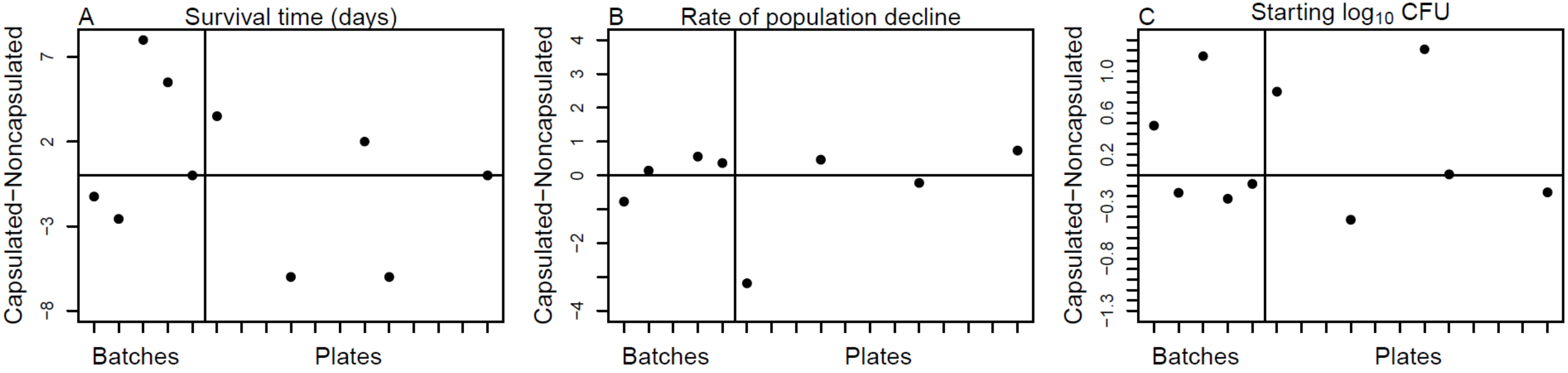

Figure S6. Capsules do not have a consistent effect on survival. A. Difference in survival time (days) between capsulated and non-capsulated *S. suis*. Above zero: capsulated bacterial survival times are longer; below zero: non-capsulated bacterial survival times are longer. B. Difference in rate of population decline between capsulated and non-capsulated *S. suis*. C. Difference in starting CFU between capsulated and non-capsulated *S. suis*. All plotting conventions are the same as used in Figure S4.
